# Neuroprotective Effects of Lutein in the Retina

**DOI:** 10.2174/138161212798919101

**Published:** 2012-01

**Authors:** Yoko Ozawa, Mariko Sasaki, Noriko Takahashi, Mamoru Kamoshita, Seiji Miyake, Kazuo Tsubota

**Affiliations:** 1 Laboratory of Retinal Cell Biology, Keio University School of Medicine, 35 Shinanomachi, Shinjuku- ku, Tokyo 160-8582, Japan; 2 Department of Ophthalmology, Keio University School of Medicine, 35 Shinanomachi, Shinjuku-ku, Tokyo 160-8582, Japan

**Keywords:** Oxidative stress, neuroprotection, lutein, retina, visual function, protein degradation, DNA damage, synaptophysin, BDNF, ubiquitin proteasome system.

## Abstract

Although a large variety of pharmaceutical therapies for treating disease have been developed in recent years, there has been little progress in disease prevention. In particular, the protection of neural tissue is essential, because it is hardly regenerated. The use of nutraceuticals for maintaining the health has been supported by several clinical studies, including cross-sectional and interventional studies for age-related macular disease. However, mechanistic evidence for their effects at the molecular level has been very limited. In this review, we focus on lutein, which is a xanthophyll type of carotenoid. Lutein is not synthesized in mammals, and must be obtained from the diet. It is delivered to the retina, and in humans, it is concentrated in the macula. Here, we describe the neuroprotective effects of lutein and their underlying molecular mechanisms in animal models of vision-threatening diseases, such as innate retinal inflammation, diabetic retinopathy, and light-induced retinal degeneration. In lutein-treated mouse ocular disease models, oxidative stress in the retina is reduced, and its downstream pathological signals are inhibited. Furthermore, degradation of the functional proteins, rhodopsin (a visual substance) and synaptophysin (a synaptic vesicle protein also influenced in other neurodegenerative diseases such as Alzheimer’s disease and Parkinson’s disease), the depletion of brain-derived neurotrophic factor (BDNF), and DNA damage are prevented by lutein, which preserves visual function. We discuss the possibility of using lutein, an antioxidant, as a neuroprotective treatment for humans.

## INTRODUCTION

Recent progress has led to the development of various kinds of chemically synthesized drugs. However, many therapeutic compounds have been also found in nature. Here, we focus on lutein, a phytochemical categorized as a carotenoid, and describe animal data that may support its future use as a neuroprotective treatment.

## 1 GENERAL INFORMATION ABOUT LUTEIN 

**Fig. (1) Chemical structure of lutein.**
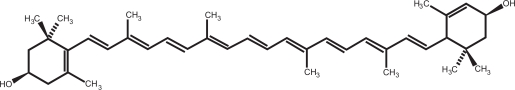
C_40_H_56_O_2_. Lutein contains double bonds that scavenge ROS.

Phytochemicals are plant-derived compounds that are not essential nutrients for sustaining life. Because they often have pigments, fragrance, or a bitter taste, they have been thought to play a protective role against external threats, such as ultraviolet light, pathogens, and creatures that eat plants. The carotenoid group includes phytochemicals with the basic structure C_40_H_56_. These compounds act as antioxidants. They contain several double bonds, which react with reactive oxygen species (ROS) to scavenge radicals. While carotenes are composed of only carbon and hydrogen, xanthophylls include other elements. Lutein is a xanthophyll that is a hydroxycarotenoid (C_40_H_56_O_2_) (Fig. **1**). Lutein’s properties are considerably different from those of carotenes. 

Carotenes are transformed to vitamin A in the body, and are therefore called pro-vitamin A [1]. Lutein is a yellow crystal that is found in some vegetables, such as kale, spinach, and broccoli, and also in the marigold flower, which is used as a source for a supplementary micronutrient.

**Fig. (2) The human retina.**
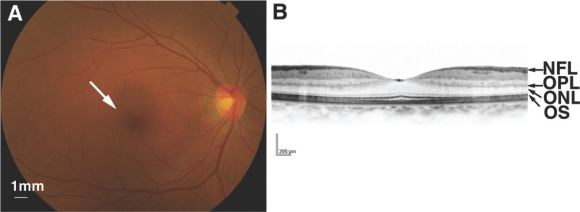
(**A**) Fundus photograph. An arrow shows the center of the retina, where photoreceptor cells and light stimuli are concentrated. (**B**) Optical coherence tomography (OCT) image showing a cross section of the central part of the retina. NFL, nerve fiber layer; OPL, outer plexiform layer; ONL, outer nuclear layer (photoreceptor cell layer); OS, outer segment.

Since animals cannot synthesize lutein, they must obtain it from the diet. It is absorbed from the intestinal epithelium into the blood, and circulates systemically to reach the liver, lung, and retina [1-3]. In the human retina, it is concentrated in the macula, the most central region (Fig.**2A, B**), so it is called a macular pigment. The analysis of stereoisomers of this macular pigment revealed two stereoisomeric carotenoids with identical properties to lutein and zeaxanthin [4]. The ratio of these isomers is different in each tissue (*e.g., *between the retina and plasma), suggesting that they are locally inter-converted. This study further showed that this transformation (3R,30R,60R)-lutein ↔ nondietary metabolite (3R,30S-meso)-zeaxanthin ↔ (3R,30R)-zeaxanthin occurs metabolically through a series of oxidation-reduction and double-bond isomerization reactions [1]. 

Lutein exists not only in the macula but also broadly in the retina [5]. It is found in the photoreceptor outer segments (OSs), where light stimuli are received, and in the retinal pigment epithelium, where OSs are phagocytosed and recycled. However, interestingly, resonance Raman imaging showed that lutein is most abundant in a neuronal network layer connecting the photoreceptor cells (the outer nuclear layer; ONL) to the secondary neurons, called the outer plexiform layer (OPL) (Fig.**2B**) [6]. Because lutein is a yellow pigmented crystal, it has long been thought to act as a filter of blue light, which has high energy of the visible spectrum.

## 2 RESULTS OF CLINICAL STUDIES

It has long been known that some phytochemicals can be medically beneficial. For example, the cardiac drug digitalis was first discovered in the leaves of the digitalis flower [7], and the analgesic morphine was found in the opium poppy [8]. However, lutein’s effects have just started to be evaluated, and only for limited use as a preventive therapy of age-related macular degeneration (AMD), a vision-threatening disease.

After the report of an inverse association between vegetable/fruit intake and AMD [9], a large clinical study, the Age-related Eye Disease Study (AREDS), was performed to examine ways to prevent AMD. In its annexed study, participants reporting the highest dietary intake of lutein/zeaxanthin were statistically less likely to have advanced AMD (either the atrophic or exudative type) or to be at high risk of developing it, than those reporting the lowest dietary intake [10], suggesting that lutein plays some protective role in the eye. Another study showed that, in 90 patients with atrophic AMD, lutein intake increased the macular pigment optical density (MPOD) and was correlated with an increase in visual contrast sensitivity [11]. The incidence of AMD (exudative type) was low in those who took a large amount of lutein or zeaxanthin [12] and who showed high levels of plasma carotenoid [13,14] but not of other antioxidants, vitamin C, E, or selenium [13].

A study using donor eyes showed a negative association between lutein content in the retina and AMD risk [15]. Retinas from 56 donors with AMD and 56 controls were analyzed using high-performance liquid chromatography. The levels of lutein and zeaxanthin were lower in the AMD retinas in both the central retina (62% of control), including the macula, and the peripheral retina (70-80% of control). Taken together, these observations indicate that lutein obtained from the diet accumulates in the retina and may act locally to prevent disease.

In a second large-scale clinical study, AREDS2, the effects of lutein/zeaxanthin and/or docosahexaenoic/eicosapentaenoic acids in approximately 4000 AMD patients are presently being tested for their possible use in preventive therapy. This possibility is based on the involvement of oxidative stress-related conditions, such as smoking and hypertension [16], in the risk for AMD, and the proposed anti-oxidative action of lutein. However, the biological role of lutein in the body, including the retina, has not yet been elucidated. The association between arthritis and AMD [16], and between the genetic abnormality of complement factor and AMD [17-19], have also been reported, suggesting that inflammation is related to AMD. However, an anti-inflammatory effect of lutein has been hardly documented. 

The retina is part of the central nervous system, and thus regenerates poorly, if at all. Therefore, it is essential to protect the retinal neurons from oxidative stress and inflammation. Many adult diseases, including diabetes and AMD, involve oxidative stress and inflammation. To investigate lutein’s potential as a preventative therapy for these diseases, it is important to study its mechanisms of action *in vivo.*

## 3 BIOLOGICAL EFFECTS AND UNDERLYING MOLECULAR MECHANISMS OF LUTEIN IN MOUSE RETINAL DISEASE MODELS 

### 1) Lutein’s Effects on Acute Innate Inflammation of the Retina

To simplify the analysis of lutein’s biological actions *in vivo*, we first used an endotoxin-induced uveitis (EIU) mouse model, generated by the injection of lipopolysaccaride (LPS) [20].

#### i) Retinal Neuronal Disorder During Inflammation

In the EIU model, the innate immune system causes intraocular inflammation, which involves the neural retina [20-24]. In human uveitis cases, inflammation spreads to the retina as well as the uvea and causes visual dysfunction, although the underlying mechanism has long been obscure. In the EIU model mice, electroretinogram (ERG), an objective method for measuring visual function in both humans and mice, shows a decrease in the a-wave amplitude, and photoreceptor cell dysfunction [20,21,23,24]. This effect involves a reduction in rhodopsin protein, which is localized in the OS of photoreceptor cells and is indispensable for photo-transduction. This protein reduction results from excessive protein degradation by the ubiquitin-proteasome system (UPS), in which the protein-selective E3-ligase, ubiquitin-protein ligase E3 component n-recognin 1 (Ubr1) [24], is upregulated by STAT3 activation downstream of inflammatory signals, such as interleukin-6 (IL-6). The loss of rhodopsin shortens the OSs [25] and decreases light reception and photoreceptor cell function. Under physiological conditions, suppressor of cytokine signaling 3 (SOCS3), which modulates activated STAT3 *via* a feedback loop, fine-tunes this signal [24,26]. Once this feedback system is overwhelmed, however, the STAT3 activation and subsequent rhodopsin reduction are accelerated, resulting in vision loss [23,24].

#### ii) Lutein’s Effect During Inflammation Includes ROS Suppression

The UPS is also accelerated by protein modifications resulting from oxidative stress. Therefore, if oxidative stress is involved in inflammation and lutein has an anti-oxidative effect *in vivo*, lutein might protect visual function by suppressing inflammation-related protein degradation.

**Fig. (3) Lutein’s effect on oxidative stress in the inflamed retina.**
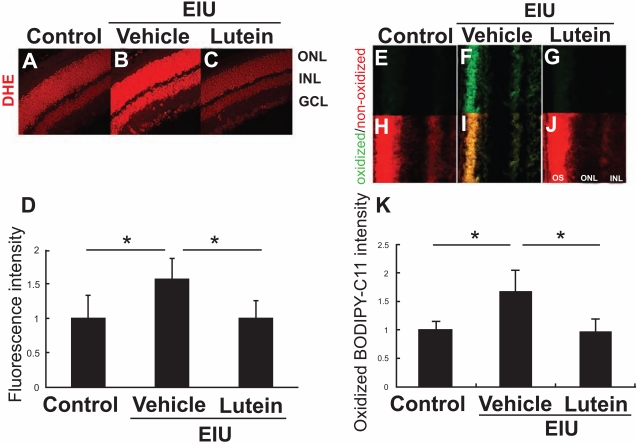
ROS in the retina were detected by dihydroethidium (DHE) (A-D) and BODIPY-C11 which indicates lipid peroxidation (E-K). Lutein administration suppresses ROS in the retina. (Sasaki *et al. Invest Ophthalmol Vis Sci* 2009).

To test this hypothesis, we pre-treated EIU model mice with lutein, and examined whether the rhodopsin protein level, OS length, and visual function (photoreceptor cell function) were preserved during inflammation [20]. Interestingly, oxidative stress was induced in the retina during inflammation, and lipid peroxidation was observed in the plasma membrane of the OSs, supporting the idea that rhodopsin, a transmembrane protein, was modified by the inflammation. These effects were successfully suppressed by lutein (Fig. **3**), indicating that lutein protects the function of the retinal neural tissue by reducing oxidative stress and protein loss. Consistently, lutein preserved a-wave amplitude in ERG, indicating that lutein protects visual function during inflammation.

#### iii) Lutein Suppresses Inflammatory Signaling

**Fig. (4) Model of the underlying mechanisms of lutein’s effect.**
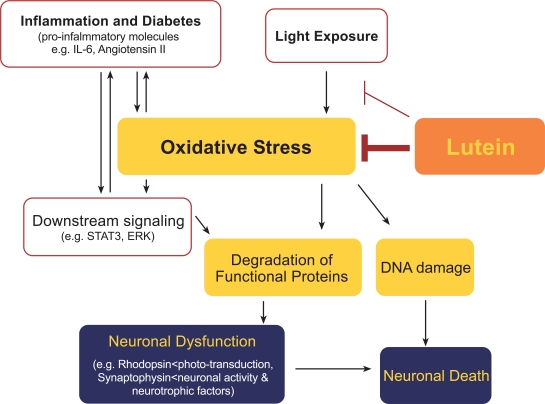
Lutein suppresses oxidative stress in tissue. This suppresses the degradation of functional proteins and DNA damage caused by pro-inflammatory molecules induced in the tissue during inflammation or diabetes, or as a result of light exposure, preventing neuronal dysfunction and death. Lutein can absorb and block light.

Lutein’s effect on rhodopsin preservation is accompanied by a suppression of STAT3 activation. STAT3 is generally activated by inflammatory cytokines, one of which, IL-6 is regulated by activated STAT3. Thus, once STAT3 activation exceeds a certain level, the IL-6-STAT3 pathway enters a vicious cycle that exacerbates the pathological condition. However, lutein suppresses STAT3 activation, thereby suppressing this vicious cycle, and efficiently preventing tissue damage. Regarding the mechanism for inhibiting the STAT3 activation, the direct pathway of STAT3 phosphorylation and activation by ROS ^27^ may be suppressed by lutein. Therefore, there is a link between oxidative stress and inflammation, which can be regulated by lutein; thus, lutein has an anti-inflammatory effect (Fig.**4**). This scenario is supported by previous reports showing that lutein reduces the intraocular infiltration of inflammatory cells in models of EIU [28] and laser-induced choroidal neovascularization [29]. 

STAT3 activation and its related pathological changes in the retina of the EIU model are also suppressed by angiotensin II type 1 receptor (AT1R) blockers [30]. This is not only because AT1R blockers suppress STAT3 activation downstream of AT1R, but also because they suppress the ROS generation by NADPH oxidase downstream of AT1R signaling.

### 2) Lutein’s Effects on Diabetic Retinopathy

Although chronic neurodegeneration is well known to occur in diabetes, there is no established therapy to prevent it. Given that diabetes involves oxidative stress, a constant intake of the anti-oxidant, lutein, might help to prevent this complication in the retina.

#### i) Retinal Neurodegeneration in Diabetes

In a mouse model of type 1 diabetes induced by injecting streptozotocin (STZ), a compound that is toxic to the insulin-producing beta cells of the pancreas, impaired visual function is obvious one month after the onset of diabetes [21]. The ERG shows impaired oscillatory potentials (OPs), which reflects inner retinal neurode-generation. This change is also observed in humans from an early stage, before diabetes-induced microangiopathy is obvious [31,32]. The retinal neurodegeneration and visual dysfunction continue to progress after microangiopathy has been made quiescent by laser treatment or surgical intervention. This neuronal disorder is caused by the diabetic and toxic microenvironment. 

We showed that local AT1R signaling contributes to this retinal neurodegeneration [21]. We found that the angiotensin II signal is upregulated locally in the retina, and the suppression of neuronal damage by AT1R blockers was not associated with a reduction in systemic blood glucose levels. Inner retinal layer involves a neuronal synaptic network where primary integration of the visual information occurs *via* active neurotransmitter release. Synaptic vesicles are indispensable for this biological activity, and synaptophysin, a synaptic-vesicle component, plays a key role in various neurodegenerative diseases, most likely through synaptic network abnormalities; genetically abnormal synaptophysin causes mental retardation [33], and abnormal forms of it are found in Alzheimer’s disease and Parkinson’s disease [34]. 

We showed that the protein level of synaptophysin is also reduced in the diabetic retina through the pro-inflammatory AT1R signaling pathway [21] by activating Extracellular Signal-regulated Kinase (ERK). ERK can induce seven in absentia (Sina) [35], which mammalian homologues, the seven in absentia homologues (Siahs), have turned out to be a synaptophysin-selective E3-ligase [36]. The involvement of a common pathogenic pathway, *i.e.,* protein degradation, in the diabetes and EIU models is interesting, and consistent with previous findings that diabetic influence in the retina involves inflammation [21,37] and oxidative stress [38]. Since locally activated AT1R signaling can cause oxidative stress, we hypothesized that a constant intake of lutein might prevent the retinal neurodegeneration induced by diabetes.

#### ii) Lutein Protects Neuronal Function in the Diabetic Retina

Strikingly, diabetic mice constantly fed a lutein-supplemented diet show preserved visual function as measured by ERG, indicating the prevention of inner retinal damage [38]. Lutein suppresses the generation of ROS in the diabetic retina without changing the systemic blood glucose level.

In the retina of lutein-fed diabetic mice, ERK activation is suppressed and synaptophysin protein is preserved. Therefore, the chronic activation of ERK is also associated with oxidative stress and is affected by lutein’s anti-oxidative and anti-inflammatory actions, to protect neuronal activity. Moreover, lutein is reported to bind to a protein, glutathione-S-transferase 1, in the monkey retina, which localizes to the synaptic region, OPL, in addition to the OSs of photoreceptor cells [6]. This distribution is consistent with lutein’s protection of retinal neurons.

Lutein’s protection of synaptic vesicle protein affects more than the synaptic activity. Neuronal activity induces the translocation of calcium ions, which needs to be regulated to maintain cellular survival and induces the expression of a neuronal trophic factor, brain-derived neurotrophic factor (BDNF) [39,40]. BDNF, whose deficiency is associated with a number of neurodegenerative disorders (*e.g.* Huntington’s disease, Alzheimer’s disease, and Parkinson’s disease), is also downregulated in the diabetic retina [38]. Importantly, however, lutein attenuates this decrease, most likely through its protection of synaptic activity (Fig.**4**). It has not been determined whether BDNF can be modified or degraded by oxidative stress. 

It is anticipated that neurodegenerative diseases will be treated using strategies designed to increase the BDNF level [40] —the administration of recombinant proteins or drugs that activate its endogenous expression and gene therapy to relieve the symptoms of Alzheimer’s disease and Parkinson’s disease are now in trial. If the BDNF reduction in these neurodegenerative diseases involves a mechanism common to that of diabetic retinopathy, lutein administration might also be considered as a therapeutic strategy for these neuronal diseases.

#### iii) Lutein Promotes Neuronal Survival in the Diabetic Retina

Reductions in the neuronal functional protein synaptophysin and in the neurotrophic factor BDNF are implicated in the neuronal dysfunction of diabetic model mice, as shown by ERG [38]. These changes are already visible in 1 month from the onset of diabetes, although the change might be reversible at this time. However, diabetic neuronal damage ultimately causes neuronal cell death; after 4 months from the onset of diabetes, the numbers of retinal ganglion cells and inner retinal cells are clearly reduced [38]. Retinal neurons do not appear to proliferate; thus, this change is irreversible. Importantly, lutein protects the retinal ganglion cells and inner retinal cells from diabetes-induced cell death for at least 4 months after diabetes onset. That lutein protects not only neuronal function but also supports cell survival in the animal model helps explain its effectiveness. Although further studies are required, the long-term constant intake of lutein is a potential neuroprotective and preventive therapy for diabetic retinopathy.

### 3) Lutein’s Effects in Light-induced Retinal Degeneration

Lutein has anti-oxidative and anti-inflammatory effects that are independent of light stimuli (described above). However, lutein’s role in the macula is assumed to be in protecting the macular region of the retina from light damage. To investigate the mechanism of lutein’s protective effect against light exposure, we used a mouse model of light-induced retinal degeneration. In this model, photoreceptor cells gradually degenerate and die in two phases: an immediate phase, and a subsequent, long-lasting phase caused by cumulative changes in enzymatic activities that are triggered by light exposure [41-43]. In this long-lasting phase, double-stranded breaks in DNA cause the loss of photoreceptor cells by apoptosis; notably, this most severe type of DNA damage is attenuated by lutein pre-treatment [44] (Fig.**4**). 

The light-induced visual dysfunction measured by ERG in these mice is significantly suppressed by lutein administration. ROS induced in the light-exposed retina are also suppressed by lutein. Although it is not yet known whether this reduction in oxidative stress is caused by the blockage of light or the scavenging of ROS, this effect of lutein is significant. Mice do not have a macula, however, this effect is consistent with the lutein’s broad distribution in the whole area of the retina as reported in the human retina (described in the section 1 and 2).

## 4 SUMMARY

Lutein is produced in plants, not mammals, but it is delivered to and accumulates in mammalian tissue. Lutein’s biological effects have long been noted, but research on its underlying molecular mechanisms has just begun. Lutein affects the pathological pathways of inflammatory cytokines, such as IL-6 and angiotensin II signaling, and prevents neurodegeneration (*e.g.,* *via* the loss of function-essential proteins and trophic factors, and *via* DNA damage) that can be induced by oxidative stress. Lutein protects tissue against pathological stimuli regardless of light exposure. Further investigations will continue to explore the use of lutein in tissue protection, including neuroprotection.

## ABBREVIATIONS

AMD: = Age-related macular degeneration;
AT1R: = Angiotensin II type 1 receptor;
BDNF: = Brain-derived neutrophic factor;
EIU: = Endotoxin-induced uveitis;
ERG: = Electroretinogram;
ERK: = Extracellular Signal-regulated Kinase;
IL: = Interleukin;
LPS: = Lipopolysaccaride;
ONL: = Outer nuclear layer;
OPL: = Outer plexiform layer;
OSs: = Outer segments;
UPS: = Ubiquitin-proteasome system

## References

[1]  Khachik F, Bernstein PS, Garland DL (1997). Identification of lutein and
zeaxanthin oxidation products in human and monkey retinas. Invest
Ophthalmol Vis Sci.

[2]  Khachik F, Carvalho L, Bernstein PS, Muir GJ, Zhao DY, Katz 
NB (2002). Chemistry distribution and metabolism of tomato carotenoids
and their impact on human health. Exp Biol Med.

[3]  Yonekura L, Nagao A (2007). Intestinal absorption of dietary carotenoids. Mol Nutr Food Res.

[4]  Bone RA, Landrum JT, Hime GW, Cains A, Zamor J (1993). Stereochemistry of the human macular carotenoids. Invest Ophthalmol Vis Sci.

[5]  Rapp LM, Maple SS, Choi JH (2000). Lutein and zeaxanthin concentrations
in rod outer segment membranes from perifoveal and peripheral
human retina. Invest Ophthalmol Vis Sci.

[6]  Bhosale P, Li B, Sharifzadeh M (2009). Purification and partial characterization of a lutein-binding protein from human retina. Biochem.

[7]  Newman RA, Yang P, Pawlus AD, Block KI (2008). Cardiac glycosides as novel cancer therapeutic agents. Mol Inter.

[8]  Ziegler J, Facchini PJ, Geissler R (2009). Evolution of morphine biosynthesis in opium poppy. Phytochem.

[9]  Seddon JM, Ajani UA, Sperduto RD (1994). Dietary carotenoids
vitamins A C and E and advanced age-related macular degeneration. Eye Disease Case-Control Study Group. Jama.

[10]  SanGiovanni JP, Chew EY, Clemons TE (2007). The relationship of
dietary carotenoid and vitamin A E and C intake with age-related
macular degeneration in a case-control study AREDS Report No
22. Arch Ophthalmol.

[11]  Richer S, Stiles W, Statkute L (2004). Double-masked placebo-controlled
randomized trial of lutein and antioxidant supplementation
in the intervention of atrophic age-related macular degeneration
the Veterans LAST study (Lutein Antioxidant Supplementation
Trial). Optometry.

[12]  Tan JS, Wang JJ, Flood V, Rochtchina E, Smith W, Mitchell P (2008). Dietary antioxidants and the long-term incidence of age-related
macular degeneration the Blue Mountains Eye Study. Ophthalmol.

[13] (1993). Antioxidant status and neovascular age-related macular degeneration. Eye Disease Case-Control Study Group. Arch Ophthalmol.

[14]  Michikawa T, Ishida S, Nishiwaki Y (2009). Serum antioxidants and
age-related macular degeneration among older Japanese. Asia Pac J
Clin Nutr.

[15]  Bone RA, Landrum JT, Mayne ST, Gomez CM, Tibor SE, 
Twaroska EE (2001). Macular pigment in donor eyes with and without
AMD: a case-control study. Invest Ophthalmol Vis Sci.

[16] (2000). Age-Related Eye Disease Study Research Group Risk factors
associated with age-related macular degeneration A case-control
study in the age-related eye disease study Age-Related Eye Disease
Study Report Number 3. Ophthalmology.

[17]  Edwards AO, Ritter R 3rd, Abel KJ, Manning A, Panhuysen C, 
Farrer LA (2005). Complement factor H polymorphism and age-related
macular degeneration. Science.

[18]  Haines JL, Hauser MA, Schmidt S (2005). Complement factor H
variant increases the risk of age-related macular degeneration. Science.

[19]  Klein RJ, Zeiss C, Chew EY (2005). Complement factor H polymorphism
in age-related macular degeneration. Science.

[20]  Sasaki M, Ozawa Y, Kurihara T (2009). Neuroprotective effect of an
antioxidant lutein during retinal inflammation. Invest Ophthalmol
Vis Sci.

[21]  Kurihara T, Ozawa Y, Nagai N (2008). Angiotensin II type 1 receptor
signaling contributes to synaptophysin degradation and neuronal
dysfunction in the diabetic retina. Diabetes.

[22]  Nagai N, Oike Y, Noda K (2005). Suppression of ocular inflammation
in endotoxin-induced uveitis by blocking the angiotensin II
type 1 receptor. Invest Ophthalmol Vis Sci.

[23]  Ozawa Y, Kurihara T, Tsubota K, Okano H (2011). Regulation of post-transcriptional modification as a possible therapeutic approach for
retinal neuroprotection. J Ophthalmol.

[24]  Ozawa Y, Nakao K, Kurihara T (2008). Roles of STAT3/SOCS3
pathway in regulating the visual function and ubiquitin-proteasome-
dependent degradation of rhodopsin during retinal inflammation. J Biol Chem.

[25]  Lem J, Krasnoperova NV, Calvert PD (1999). Morphological
physiological and biochemical changes in rhodopsin knockout
mice. Proc Natl Acad Sci USA.

[26]  Ozawa Y, Nakao K, Shimazaki T (2007). SOCS3 is required to temporally
fine-tune photoreceptor cell differentiation. Dev Biol.

[27]  Madamanchi NR, Li S, Patterson C, Runge MS (2001). Reactive oxygen
species regulate heat-shock protein 70 *via* the JAK/STAT pathway. Arterioscler Thromb Vasc Biol.

[28]  Jin XH, Ohgami K, Shiratori K (2006). Inhibitory effects of lutein on
endotoxin-induced uveitis in Lewis rats. Invest Ophthalmol Vis Sci.

[29]  Izumi-Nagai K, Nagai N, Ohgami K (2007). Macular pigment lutein
is antiinflammatory in preventing choroidal neovascularization. Arterioscler
Thromb Vasc Biol.

[30]  Kurihara T, Ozawa Y, Shinoda K (2006). Neuroprotective effects of
angiotensin II type 1 receptor (AT1R) blocker telmisartan *via*
modulating AT1R and AT2R signaling in retinal inflammation. Invest
Ophthalmol Vis Sci.

[31]  Kizawa J, Machida S, Kobayashi T, Gotoh Y, Kurosaka D (2006). Changes of oscillatory potentials and photopic negative response in
patients with early diabetic retinopathy. Jpn J Ophthalmol.

[32]  Yonemura D, Tsuzuki K, Aoki T (1962). Clinical importance of the oscillatory
potential in the human ERG. Acta Ophthalmologica.

[33]  Tarpey PS, Smith R, Pleasance E (2009). A systematic, large-scale
resequencing screen of X-chromosome coding exons in mental retardation. Nat Genet.

[34]  Zhan SS, Beyreuther K, Schmitt HP (1993). Quantitative assessment of the
synaptophysin immuno-reactivity of the cortical neuropil in various
neurodegenerative disorders with dementia. Dementia (Basel
Switzerland).

[35]  Carthew RW, Neufeld TP, Rubin GM (1994). Identification of genes that
interact with the sina gene in Drosophila eye development. Proc
Natl Acad Sci USA.

[36]  Wheeler TC, Chin LS, Li Y, Roudabush FL, Li L (2002). Regulation of
synaptophysin degradation by mammalian homologues of seven in
absentia. J Biol Chem.

[37]  Nagai N, Izumi-Nagai K, Oike Y (2007). Suppression of diabetes-induced
retinal inflammation by blocking the angiotensin II type 1
receptor or its downstream nuclear factor-kappaB pathway. Invest
Ophthalmol Vis Sci.

[38]  Sasaki M, Ozawa Y, Kurihara T (2010). Neurodegenerative influence
of oxidative stress in the retina of a murine model of diabetes. Diabetologia.

[39]  Binder DK, Scharfman HE (2004). Brain-derived neurotrophic factor. Growth factors.

[40]  Kohara K, Kitamura A, Morishima M, Tsumoto T (2001). Activity-dependent transfer of brain-derived neurotrophic factor to postsynaptic
neurons. Science.

[41]  Cortina MS, Gordon WC, Lukiw WJ, Bazan NG (2003). DNA repair in photoreceptor survival. Mol Neurobiol.

[42]  Gordon WC, Casey DM, Lukiw WJ, Bazan NG (2002). DNA damage and repair in light-induced photoreceptor degeneration. Invest Ophthalmol Vis Sci.

[43]  Specht S, Leffak M, Darrow RM, Organisciak DT (1999). Damage to rat retinal DNA induced *in vivo* by visible light. Photochem Photobiol.

[44]  Sasaki M, Yuki K, Kurihara T (2011). Biological role of lutein in the light-induced retinal degeneration. J Nutr Biochem.

